# The dataset of the CLU lichen herbarium (Calabria, Italy)

**DOI:** 10.3897/BDJ.12.e116965

**Published:** 2024-03-08

**Authors:** Matteo Conti, Stefano Martellos, Andrea Moro, Pier Luigi Nimis, Domenico Puntillo

**Affiliations:** 1 Dept. Of Life Sciences, University of Trieste, Trieste, Italy Dept. Of Life Sciences, University of Trieste Trieste Italy; 2 Museo di Storia Naturale della Calabria ed Orto Botanico, Arcavacata di Rende (Cosenza), Italy Museo di Storia Naturale della Calabria ed Orto Botanico Arcavacata di Rende (Cosenza) Italy

**Keywords:** occurrence, Calabria, Italy, lichenised fungi, specimen

## Abstract

**Background:**

Calabria, the southernmost tip of the Italian Peninsula, is a biogeographically very interesting region for lichenologists, characterised by the abundance of oceanic and suboceanic species with subtropical affinities, but also by the presence of the southernmost outposts of several boreal species on the highest peaks. The lichen biota of Calabria, which began to be intensively studied only from the 1980s, hosts more than 1000 infrageneric taxa. The lichen herbarium of the Botanical Garden of the University of Calabria (CLU) is the most relevant lichen collection from this region. It was established in 1985 and it presently includes 16926 specimens, most of which were collected in Calabria, although there are also several specimens from other parts of Italy and from abroad.

**New information:**

This dataset contains 16926 records of lichens for a total of 1316 species. Of the 15219 georeferenced specimens, 10254 were collected in Calabria, while 4965 in other administrative regions of Italy. The dataset is available through GBIF, as well as in ITALIC, the Information System of Italian Lichens.

## Introduction

Calabria, the southernmost tip of the Italian Peninsula, is a biogeographically very interesting region for lichenologists. The rugged morphology, the variety of substrata, the suboceanic climate conditions, the abundance of old forests and ancient cultivations of olive groves and the weak industrial development are responsible for a rich and varied lichen biota (Nimis 1993), characterised by the abundance of oceanic and suboceanic species with subtropical affinities, but also by the presence of the southernmost outposts of several boreal species on the highest peaks (Bartoli et al. 1991, Incerti and Nimis 2006).

The first scattered lichen records from Calabria were contained in a series of papers devoted to the lichens of southern Italy by A. Jatta (1852-1912), after which the lichen biota of Calabria remained virtually unexplored for almost a century (see Nimis 1993). It was only after the foundation of the Italian Lichen Society in the 1980s century that lichenological studies in Calabria started to flourish again, thanks to the activity of researchers at the Botanical Institute of the University of Calabria in Cosenza, who published a series of florulas of selected areas (Puntillo 1987, Puntillo 1993, Puntillo 1995, Puntillo and Puntillo 2004, Puntillo 2011), taxonomic revisions of difficult groups such as the Caliciales (Puntillo 1994, Puntillo and Puntillo 2009), the Pannariaceae (Codogno and Puntillo 1991, Codogno and Puntillo 1993), the Umbilicariaceae (Codogno and Puntillo 1989) and foliicolous lichens (Puntillo and Vězda 1994, Puntillo 2000) and descriptions of new species (Codogno et al. 1989, Nimis and Poelt 1989, Puntillo and Vězda 1991, Diederich and Puntillo 1995, Puntillo and Ravera 2013). The first checklist of the lichens of Calabria (Puntillo 1996) listed 629 infrageneric taxa, a number which rapidly rose to 900 in 2003 (Nimis and Martellos 2003), 960 in 2016 (Nimis 2016), 992 in 2022 and 1037 in 2023 (Nimis 2023).

The lichen herbarium of the Botanical Garden of the University of Calabria (CLU) is the most relevant lichen collection from this region. It was first established in 1985 (Codogno and Puntillo 1990) and currently includes 16926 specimens, most of which were collected in Calabria. CLU also contains several specimens from other parts of Italy and abroad, the latter mainly due to exchange of exsiccata with some important lichenological centres such as the University of Graz (Josef Poelt) and the Czechoslovak Academy of Sciences in Brno (Prof. A. Vězda).

The digitisation and publication of the CLU lichen herbarium was carried out in the framework of the Dryades project (Nimis et al. 2003). This initiative focuses on making modern lichen herbaria in Italy, specifically those with specimens collected after 1950, publicly accessible on ITALIC, the Information System on Italian Lichens (Nimis 2023) and on the Global Biodiversity Information Facility (GBIF 2023).

## Sampling methods

### Study extent

Most specimens (61% of the total) were collected in Calabria, although there are also several specimens from other parts of Italy and exsiccata from international herbaria.

### Sampling description

Specimen labels were digitised in a spreadsheet and standardised to comply with the Darwin Core (Wieczorek et al. 2012). Subsequently, the data were imported into a MySQL database and published on ITALIC (Nimis 2023) and GBIF (2023).

Due to the absence of geographic coordinates in the specimen labels, localities were georeferenced a posteriori (only Italian localities) combining Google Maps and QGIS (2023). The point-radius method was employed to determine both the coordinates and the associated uncertainty, adhering to the best georeferencing practices by Chapman and Wieczorek (2020). To enhance the precision of the georeferencing in Calabria, where the majority of samples have been collected, regional maps sourced from the Geoportale della regione Calabria (2023) have been consulted.

### Quality control

The dataset includes specimens from taxonomically critical groups. To ensure the quality of the data, specimens were sent to specialists who revised the identification. The scientific names originally written on the specimen labels have been transcribed in the verbatimIdentification field. The currently accepted names, aligned with the most recent version of the Checklist of the Lichens of Italy (Nimis 2016) using the name match tool in ITALIC (Martellos et al. 2023), were reported in the scientificName field.

## Geographic coverage

### Description

The dataset contains 15219 georeferenced records (90% of the total) (Puntillo et al. 2023). Of these records, 10254 specimens were collected in Calabria while 4965 in other administrative regions of Italy: Campania (1959), Sicilia (829), Basilicata (582), Toscana (267), Friuli Venezia Giulia (263), Sardegna (249), Lombardia (189), Puglia (177), Lazio (124), Umbria (86), Valle d'Aosta (71), Veneto (51), Trentino-Alto Adige (31), Emilia-Romagna (26), Piemonte (18), Abruzzo (17), Marche (13) and Molise (13).

**Figure 1.**
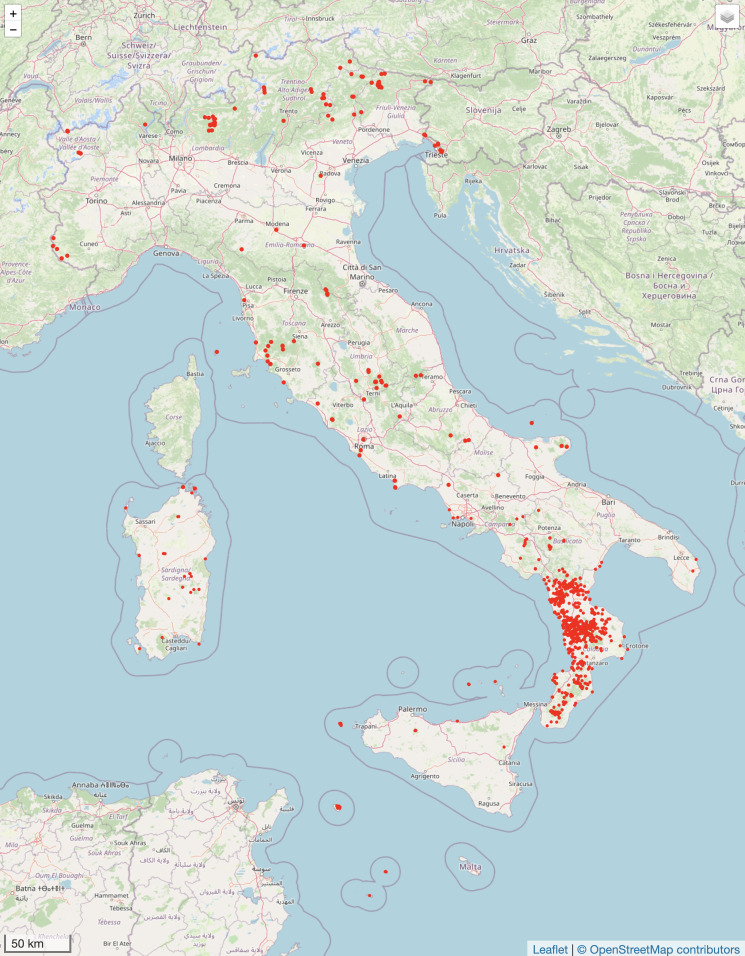
Distribution map of CLU Herbarium specimens in Italy. Map created with Leafletjs (Agafonkin 2023).

The geographic distribution of the specimens is depicted in Fig. 1.

### Coordinates

35.516 and 46.809 Latitude; 6.889 and 18.432 Longitude.

## Taxonomic coverage

### Description

Specimens in the CLU lichen herbarium belong to 1316 species, 465 genera, 124 families, 55 orders and 11 classes. Amongst these, 64 species (4.86% of the total) are non-lichenised fungi. The most represented familes and genera are shown in Table 1 and Table 2, while the number of taxa and specimens for each class, order, family and genus are provided in tabular format (Suppl. material 1) and as a Krona graph (Suppl. material 2, Ondov et al. 2011).

## Temporal coverage

### Notes

**Figure 2.**
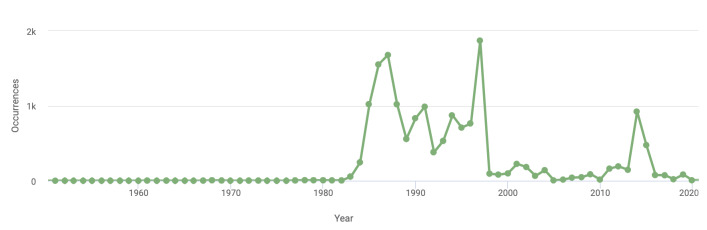
Lichens occurrences per year.

The CLU lichen herbarium was first established in 1985. The few specimens dated before 1985 come from exchanges with other herbaria or from exsiccata collections. The temporal distribution of the dataset is shown in Fig. 2.

## Usage licence

### Usage licence

Creative Commons Public Domain Waiver (CC-Zero)

### IP rights notes

This work is licensed under a Creative Commons Attribution (CC-BY 4.0) License.

## Data resources

### Data package title

CLU Lichen Herbarium

### Resource link

https://doi.org/10.15468/qyn762

### Alternative identifiers

256cae46-976d-4cf9-addf-82e5ccd9effc; https://cloud.gbif.org/eca/resource?r=clu

### Number of data sets

1

### Data set 1.

#### Data set name

CLU Lichen Herbarium

#### Data format

Darwin Core

#### Download URL

https://www.gbif.org/occurrence/download?dataset_key=256cae46-976d-4cf9-addf-82e5ccd9effc

#### Description

The lichen herbarium was started in 1985 and currently includes ca. 17,000 samples, collected mainly by D. Puntillo, in various parts of Italy, especially in Calabria. Several groups have been revised by specialists.

**Data set 1.** 

Column label	Column description
occurrenceID	A unique identifier for each occurrence record in the dataset.
type	The nature of the occurrence record.
language	Language used for the resource.
licence	Terms under which the dataset is made available.
institutionID	Unique identifier for the institution holding the specimens.
institutionCode	Acronym representing the institution.
datasetName	Title of the dataset.
basisOfRecord	The basis on which the record is made.
recordedBy	Individuals responsible for creating the occurrence record.
eventDate	Date on which the occurrence was recorded.
continent	Name of the continent where the occurrence was recorded.
country	Name of the country where the occurrence was recorded.
countryCode	Standardised code representing the country.
locality	Locality where the occurrence was recorded.
minimumElevationInMetres	Minimum elevation at which the occurrence was recorded.
maximumElevationInMetres	Maximum elevation at which the occurrence was recorded.
decimalLatitude	Latitude of the location in decimal degrees.
decimalLongitude	Longitude of the location in decimal degrees.
geodeticDatum	Reference ellipsoid used for specifying geographic coordinates.
coordinateUncertaintyInMetres	Uncertainty associated with the geographic coordinates.
verbatimIdentification	Original identification, as reported on the specimens label.
scientificName	Scientific name of the organism.
kingdom	Taxonomic kingdom to which the organism belongs.
taxonRank	The taxonomic rank of the most specific name.
stateProvince	Administrative region where the occurrence was recorded.

## Supplementary Material

AC11A1B1-BAEF-530A-A9DE-9A704304174B 10.3897/BDJ.12.e116965.suppl1 Supplementary material 1 Table of specimens and taxa in the dataset Data type Table File: oo_947961.tsv https://binary.pensoft.net/file/947961 Matteo Conti

77537B28-30B3-5A4C-B6DD-5F015797A68E 10.3897/BDJ.12.e116965.suppl2 Supplementary material 2 Krona graph of specimens and taxa in the dataset Data type HTML file File: oo_947968.html https://binary.pensoft.net/file/947968 Matteo Conti

## Figures and Tables

**Table 1.** Most represented families in the dataset.

Family	Number of specimens	Number of species
Parmeliaceae	1583	122
Byssolomataceae	1520	56
Caliciaceae	1087	40
Ramalinaceae	996	99
Coniocybaceae	945	17
Teloschistaceae	726	82
Porinaceae	711	26
Physciaceae	667	62
Arthoniaceae	580	46
Cladoniaceae	508	58

**Table 2.** Most represented genera in the dataset.

Genus	Number of specimens	Number of species
Chaenotheca	882	15
Calicium	854	16
Byssoloma	767	9
Fellhanera	580	8
Cladonia	503	55
Porina	455	14
Ramalina	360	26
Opegrapha	357	11
Pseudosagedia	355	9
Umbilicaria	273	19
