# Supplementary material for: The dataset of the CLU lichen herbarium (Calabria, Italy)
Source: Biodivers Data J. 2024 Mar 8;12:e116965. doi: 10.3897/BDJ.12.e116965 (PMC10944559; doi:10.3897/BDJ.12.e116965)
Supplement: Supplementary material 2 — Krona graph of specimens and taxa in the dataset [file bdj-12-e116965-s002.html]

Javascript must be enabled to view this page.

magnitude

CLU taxa
CLU specimens

 1502
 16926

 1502
 16926

 1472
 16832

 118
 1574

 110
 1556

 49
 580

 31
 258

 3
 10

 4
 91

 2
 97

 1
 1

 1
 64

 1
 2

 1
 2

 1
 14

 1
 2

 1
 20

 1
 18

 1
 1

 2
 29

 2
 29

 1
 1

 1
 1

 10
 119

 6
 61

 2
 50

 2
 8

 13
 365

 1
 8

 12
 357

 34
 460

 3
 31

 1
 66

 4
 26

 6
 188

 3
 15

 4
 7

 1
 2

 1
 15

 1
 22

 7
 60

 2
 20

 1
 8

 1
 2

 1
 2

 8
 18

 8
 18

 7
 17

 1
 1

 10
 150

 10
 150

 9
 148

 1
 57

 7
 90

 1
 1

 1
 2

 1
 2

 17
 945

 17
 945

 17
 945

 15
 882

 2
 63

 97
 835

 3
 9

 3
 9

 3
 9

 2
 16

 1
 4

 1
 4

 1
 12

 1
 12

 2
 5

 2
 5

 1
 4

 1
 1

 4
 4

 4
 4

 1
 1

 3
 3

 3
 4

 3
 4

 3
 4

 1
 12

 1
 12

 1
 12

 2
 22

 1
 1

 1
 1

 1
 21

 1
 21

 6
 14

 6
 14

 6
 14

 2
 3

 2
 3

 2
 3

 2
 58

 1
 54

 1
 54

 1
 4

 1
 4

 10
 136

 10
 136

 6
 106

 3
 29

 1
 1

 9
 39

 8
 11

 2
 3

 6
 8

 1
 28

 1
 28

 2
 7

 2
 7

 2
 7

 22
 142

 1
 3

 1
 3

 2
 10

 1
 6

 1
 4

 3
 37

 1
 2

 2
 35

 4
 20

 2
 8

 2
 12

 8
 63

 1
 2

 5
 35

 2
 26

 4
 9

 4
 9

 18
 337

 18
 337

 1
 45

 1
 4

 1
 2

 1
 2

 2
 5

 9
 268

 3
 11

 8
 26

 3
 6

 1
 4

 2
 2

 5
 20

 1
 9

 4
 11

 1
 1

 1
 1

 1
 1

 97
 844

 2
 5

 1
 3

 1
 3

 1
 2

 1
 2

 24
 271

 20
 220

 2
 4

 11
 181

 1
 16

 3
 12

 3
 7

 4
 51

 1
 2

 3
 49

 1
 3

 1
 3

 1
 3

 13
 152

 13
 152

 1
 8

 12
 144

 5
 7

 5
 7

 5
 7

 52
 406

 52
 406

 2
 19

 1
 7

 3
 10

 6
 77

 1
 1

 4
 65

 4
 29

 1
 3

 1
 1

 2
 3

 1
 7

 1
 34

 1
 7

 1
 29

 1
 7

 2
 12

 3
 17

 1
 1

 4
 9

 1
 43

 3
 7

 8
 18

 20
 131

 15
 111

 15
 111

 1
 27

 1
 2

 7
 62

 4
 15

 1
 2

 1
 3

 4
 18

 4
 18

 4
 18

 1
 2

 1
 2

 1
 2

 1069
 12159

 23
 131

 23
 131

 16
 95

 1
 6

 2
 9

 2
 8

 2
 13

 1
 1

 1
 1

 1
 1

 17
 143

 3
 28

 2
 27

 1
 1

 10
 83

 1
 7

 1
 2

 1
 22

 1
 27

 6
 25

 4
 32

 4
 32

 102
 1754

 40
 1087

 3
 90

 1
 4

 10
 53

 16
 854

 2
 37

 4
 25

 2
 3

 1
 20

 1
 1

 62
 667

 2
 75

 1
 4

 1
 3

 1
 1

 1
 45

 1
 13

 1
 12

 9
 52

 13
 230

 3
 8

 6
 51

 2
 71

 20
 99

 1
 3

 18
 199

 18
 199

 12
 166

 1
 4

 2
 25

 3
 4

 2
 2

 2
 2

 2
 2

 468
 5269

 1
 1

 1
 1

 1
 23

 1
 23

 57
 1520

 1
 10

 1
 3

 1
 1

 1
 2

 9
 767

 6
 18

 3
 6

 8
 580

 2
 46

 1
 3

 1
 1

 1
 1

 11
 62

 1
 2

 3
 6

 1
 3

 6
 9

 3
 6

 3
 6

 12
 76

 6
 42

 1
 9

 5
 25

 58
 508

 1
 1

 55
 503

 1
 2

 1
 2

 1
 7

 1
 7

 57
 261

 1
 14

 2
 7

 7
 15

 20
 115

 7
 46

 1
 2

 1
 1

 2
 3

 9
 26

 3
 9

 1
 2

 1
 12

 1
 8

 1
 1

 122
 1583

 2
 14

 1
 1

 2
 4

 1
 4

 4
 95

 1
 1

 6
 65

 1
 4

 1
 14

 1
 2

 1
 1

 3
 102

 1
 41

 1
 2

 8
 94

 3
 4

 1
 1

 1
 4

 1
 14

 2
 5

 3
 51

 3
 25

 1
 2

 3
 37

 1
 4

 1
 2

 6
 172

 4
 136

 3
 42

 12
 136

 2
 3

 1
 61

 1
 14

 4
 40

 3
 13

 1
 75

 4
 25

 1
 3

 18
 174

 1
 5

 1
 25

 6
 66

 2
 10

 2
 10

 12
 73

 1
 4

 1
 1

 4
 21

 6
 47

 102
 996

 1
 1

 15
 199

 12
 165

 4
 5

 1
 4

 3
 10

 1
 3

 3
 10

 3
 9

 2
 16

 1
 3

 7
 42

 2
 28

 2
 7

 1
 4

 27
 360

 7
 72

 7
 49

 2
 8

 1
 1

 1
 1

 1
 1

 3
 19

 3
 19

 2
 17

 1
 2

 1
 15

 30
 135

 10
 34

 7
 50

 13
 51

 2
 3

 2
 3

 2
 30

 2
 30

 24
 72

 22
 65

 1
 11

 4
 11

 2
 3

 7
 19

 1
 2

 1
 2

 5
 16

 1
 1

 1
 2

 1
 2

 1
 5

 1
 5

 114
 1659

 10
 135

 10
 135

 36
 316

 1
 1

 5
 10

 3
 13

 2
 9

 1
 10

 4
 9

 7
 232

 2
 3

 5
 19

 1
 1

 1
 2

 1
 1

 3
 6

 20
 260

 2
 5

 10
 86

 1
 2

 2
 121

 3
 11

 1
 23

 1
 12

 3
 23

 3
 23

 26
 819

 14
 455

 9
 355

 3
 9

 1
 2

 1
 2

 11
 68

 2
 3

 1
 1

 1
 1

 2
 11

 2
 25

 3
 27

 7
 36

 1
 2

 2
 3

 1
 2

 3
 29

 116
 1163

 1
 1

 1
 1

 41
 357

 1
 8

 1
 2

 7
 173

 5
 32

 5
 27

 7
 62

 1
 2

 1
 3

 13
 48

 1
 65

 1
 65

 22
 217

 1
 64

 1
 8

 1
 2

 7
 18

 3
 96

 7
 21

 2
 8

 2
 15

 1
 9

 1
 6

 6
 44

 6
 44

 21
 267

 2
 4

 5
 16

 5
 27

 3
 54

 2
 143

 1
 1

 2
 19

 1
 3

 17
 169

 14
 146

 3
 23

 4
 25

 4
 25

 1
 3

 1
 3

 49
 542

 1
 2

 1
 2

 7
 22

 2
 3

 1
 11

 2
 4

 2
 4

 9
 86

 2
 41

 1
 4

 2
 13

 3
 13

 1
 15

 3
 12

 3
 12

 7
 19

 7
 19

 21
 397

 6
 103

 12
 199

 3
 95

 1
 4

 1
 4

 17
 82

 16
 81

 16
 81

 1
 1

 1
 1

 1
 2

 1
 2

 1
 2

 88
 745

 1
 15

 1
 15

 2
 4

 2
 4

 85
 726

 5
 20

 6
 25

 1
 1

 3
 9

 14
 208

 1
 4

 6
 18

 1
 11

 2
 54

 7
 45

 6
 21

 1
 3

 4
 18

 1
 13

 1
 8

 1
 1

 1
 1

 2
 3

 2
 23

 3
 9

 2
 4

 1
 9

 1
 13

 2
 6

 4
 17

 1
 1

 1
 5

 5
 176

 29
 395

 3
 9

 3
 9

 1
 17

 1
 17

 3
 61

 3
 61

 22
 308

 3
 35

 19
 273

 8
 30

 7
 26

 4
 18

 1
 1

 2
 7

 1
 10

 1
 5

 1
 5

 2
 3

 2
 3

 1
 4

 1
 4

 1
 4

 19
 112

 19
 112

 1
 1

 1
 1

 16
 89

 2
 28

 1
 2

 1
 2

 2
 11

 1
 20

 1
 2

 1
 2

 1
 2

 1
 5

 1
 3

 1
 2

 2
 8

 1
 2

 2
 22

 2
 22

 17
 52

 1
 2

 1
 2

 1
 2

 14
 40

 10
 17

 1
 3

 2
 3

 2
 3

 4
 7

 1
 1

 2
 17

 1
 16

 1
 1

 2
 6

 1
 5

 1
 1

 1
 1

 1
 1

 1
 1

 1
 9

 1
 9

 1
 9

 18
 76

 12
 45

 6
 14

 6
 14

 2
 6

 2
 5

 2
 3

 1
 3

 1
 3

 1
 3

 1
 2

 1
 2

 1
 2

 4
 26

 4
 26

 2
 18

 1
 1

 1
 7

 6
 31

 1
 20

 1
 20

 1
 20

 5
 11

 1
 2

 1
 2

 4
 9

 4
 9

 12
 18

 12
 18

 12
 18

 12
 18

 12
 18
